# Neuroimaging evaluation of deep brain stimulation in the treatment of representative neurodegenerative and neuropsychiatric disorders

**DOI:** 10.1186/s42234-021-00065-9

**Published:** 2021-03-30

**Authors:** Shichun Peng, Vijay Dhawan, David Eidelberg, Yilong Ma

**Affiliations:** Center for Neurosciences, Institute of Molecular Medicine, The Feinstein Institutes for Medical Research, 350 Community Drive, Manhasset, New York, 11030 USA

**Keywords:** Multimodality neuroimaging, PET, SPECT, MRI, DTI, Deep brain stimulation, Vagus nerve stimulation, Parkinson’s disease, Alzheimer’s disease, Obsessive compulsive disorder, Depression, Epilepsy, Bioelectronic medicine

## Abstract

Brain stimulation technology has become a viable modality of reversible interventions in the effective treatment of many neurological and psychiatric disorders. It is aimed to restore brain dysfunction by the targeted delivery of specific electronic signal within or outside the brain to modulate neural activity on local and circuit levels. Development of therapeutic approaches with brain stimulation goes in tandem with the use of neuroimaging methodology in every step of the way. Indeed, multimodality neuroimaging tools have played important roles in target identification, neurosurgical planning, placement of stimulators and post-operative confirmation. They have also been indispensable in pre-treatment screen to identify potential responders and in post-treatment to assess the modulation of brain circuitry in relation to clinical outcome measures. Studies in patients to date have elucidated novel neurobiological mechanisms underlying the neuropathogenesis, action of stimulations, brain responses and therapeutic efficacy. In this article, we review some applications of deep brain stimulation for the treatment of several diseases in the field of neurology and psychiatry. We highlight how the synergistic combination of brain stimulation and neuroimaging technology is posed to accelerate the development of symptomatic therapies and bring revolutionary advances in the domain of bioelectronic medicine.

## Background

Functional and structural brain imaging modalities and related analytical techniques have become the cornerstone of modern neurosciences and revolutionized translational medicine in neurological and psychiatric disorders. Multi-tracer positron emission tomography (PET) or single photon emission tomography (SPECT) permits not only molecular imaging of neurotransmitters, neuroreceptors and protein aggregates, but also precise mapping of brain glucose metabolism cerebral blow flow (CBF). Non-invasive multi-spectral magnetic resonance imaging (MRI) allows for high resolution visualization and measurement of macrostructural brain anatomy, microstructural integrity with diffusion tensor imaging (DTI), CBF with arterial spin label (ASL) and blood-oxygen-level-dependent (BOLD) neural activity with rest-state functional MRI (rsfMRI). Brain anatomy is also frequently evaluated clinically with computerized tomography (CT). While direct molecular imaging modality with PET/SPECT is more specific in describing regional neuropathogenesis and confirming disease diagnosis from the objective basis of neurochemistry, it is costly in general and only available in specialty medical centers. On the other hand, indirect imaging modality is more economical and easily accessible, and able to define specific, widely-distributed brain networks and pathways associated with each disorder. The latter is concerned primarily with brain metabolism or perfusion with PET/SPECT and multiple anatomical or functional markers with MRI. Such information is highly important in facilitating early differential diagnosis, monitoring disease progression, assessing treatment effects and predicting functional recovery of novel experimental therapies.

Neuromodulation therapy has been a central technique in the non-pharmacological treatments of medically refractory patients with a variety of neurological and psychiatric disorders. There are two general classes of intervention based on deep brain stimulation (DBS) or non-invasive brain stimulation (NIBS). The effectiveness of brain stimulation strategy is dependent critically upon rigorous neuroimaging studies to help select neuroanatomical targets, screen trial participants, and evaluate postoperative safety issues and brain correlates in response to functional restorative therapies. Neuroimaging of large-scale brain networks can help clinicians to optimize target selection and improve the clinical efficacy of NIBS in neuropsychiatric diseases (Sale et al., 2015, Tremblay et al., 2020). In this review, we summarize recent advances in neuroimaging studies of DBS in the treatment of several common brain disorders following a search on PubMed of the literature published over the last decade. Table 1 provides a list of 20 studies of interest including different targets and stimulation parameters such as frequency, pulse width and voltage. Our aim is to shed new lights on the potential contributions of large-scale brain networks in further development of brain stimulation therapeutics for symptomatic treatment.

**Table 1:** Clinical characteristics and primary imaging outcomes of deep brain stimulation therapies in select neurodegenerative and psychiatric disorders

Imaging studies	Samples	Disorder	Target	Frequency (Hz)	Pulse width (μs)	Amplitude (V)	Duration (months)	Modality	Treatment-induced imaging outcomes of interest
Mure et al., 2011	9	PD	Vim	160 ± 24	100 ± 42	3.0 ± 0.6	NA	FDG PET	Decreased activity of tremor-related pattern with metabolic increase in the cerebellum/dentate nucleus and primary motor cortex, and the caudate/putamen.
	9	PD	STN	165 ± 30	78 ± 19	3.1 ± 0.6	NA	FDG PET	Decreased activity of motor-related pattern with metabolic abnormality; decreased activity of the tremor-related pattern but to a less degree than with Vim DBS.
Horn et al., 2019	20	PD	STN	NA	NA	NA	30 ± 21	rsfMRI	Increased overall functional connectivity in the motor network by enhancing the thalamo-cortical connectivity while reducing the striatal control over basal ganglia and cerebellum.
Furukawa et al., 2020	21	PD	STN	136 ± 12	63 ± 9	2.7 ± 0.6	3–6	IMP SPECT	CBF decreased in the prefrontal and cingulate cortex, but increased in the left angular/supramarginal gyrus and cerebellum. Decreased CBF in the middle cingulate or supplementary motor cortices associated with declined drawing performance after DBS.
Gratwicke et al., 2018	6	PDD	NBM	20	60	1.5–3.0	1.5	rsfMRI	No differences in changes of functional connectivity in the default mode network between the active and sham DBS.
Gratwicke et al., 2020	6	DLB	NBM	20	60	2.0–3.0	1.5	rsfMRI	Differences of functional connectivity in the default mode network and the fronto-parietal network seen between the active and sham DBS in four patients but not significant with the random-effect model.
Maltete et al., 2021	6	DLB	NBM	20–100	60–90	2.5–3.0	3	FDG PET	Increased metabolism in the superior lingual gyrus with the active versus sham DBS.
Kuhn et al., 2015	6	AD	NBM	10–20	90–150	2.0–4.5	12	FDG PET	Increased temporal, parietal and amygdalo-hippocampal metabolism in four patients at the trend-levels.
Smith et al., 2012	5	AD	Fornix	130	90	3.0–3.5	12	FDG PET	Increased metabolism in a frontal-tempo-parieto-striato-thalamic network and a fronto-temporo-parieto-occipito-hippocampal network correlating with clinical outcome measures.
Sankar et al., 2015	6	–	–	–	–	–	–	T1 MRI	Slower rate of hippocampal atrophy with its volume changes correlated strongly with changes in hippocampal metabolism, and volume changes in the fornix and mammillary bodies.
Lozano et al., 2016	42	AD	Fornix	130	90	3	12	FDG PET	Increased metabolism in the temporo-parietal cortex, hippocampus, cuneus, and cerebellum at 6 months but not 12 months.
Le Jeune et al., 2010	10	OCD	STN	130	60	< 4.0	3	FDG PET	Decreased metabolism in the left cingulate and frontal medial gyri with the medial prefrontal and orbitofrontal changes correlating with improved OCD symptoms.
Suetens et al., 2014	16	OCD	VC/VS	100–130	210–450	4.0–10.5	1–4	FDG PET	Decreased metabolism in the anterior cingulate and the prefrontal/orbitofrontal cortices with the occipital metabolic changes correlating with improved OCD symptoms.
Dougherty et al., 2016	6	OCD	VC/VS	135	90	4.0	NA	H_2_O PET	Increased CBF in the dorsal anterior cingulate cortex correlating with improved depressive symptoms (ventral contact); Increased CBF in the thalamus, putamen and pallidum (dorsal contact).
Baldermann et al., 2019	22	OCD	ALIC/NAC	NA	NA	NA	12	DTI MRI	Greater anatomic connectivity between stimulation sites and medial/lateral prefrontal cortex reliably predicing improvement in OCD symptoms. A frontothalamic pathway underlying favorable clinical outcome.
Fridgeirsson et al., 2020	10	OCD	ALIC	130–185	90–150	3.5–6.2	> 12	rsfMRI	Decreased amygdala-insula functional connectivity correlating with improvement in mood and anxiety following DBS. DBS increased the effect of the ventromedial prefrontal cortex on the amygdala, and decreased the effect of the amygdala on the insula.
Kosel et al., 2011	15	MD	VNS	20	500	Output current: 1.2 ± 0.4 mA Stim cycles: 30 s / 5 min	2.5	HMPAO SPECT	CBF decreased in the right posterior cingulate area, the lingual gyrus and the left insula, but increased in the left dorsolateral/ventrolateral prefrontal cortex.
Conway et al., 2013	13	MD	VNS	21.5 ± 3.8	332 ± 142	Output current: 1.2 ± 0.4 mA Stim cycles: 30 s / 5 min	3–12	FDG PET	Decreased metabolism in the right rostral cingulate and DLPFC at 3 months versus baseline; increased metabolism in the substantia nigra at 12 months.
Yu et al., 2018	61	Epilepsy	VNS	NA	NA	Output current: 1.2 ± 0.4 mA Stim cycles: 30 s / 5 min	49 ± 33	FDG PET	Decreased preoperative metabolic connectivity in the brainstem, cerebellum, putamen, cingulate gyrus and insula in the responders 12 months after VNS.
Ibrahim et al., 2017	29	Epilepsy	VNS	NA	NA	NA	12	rsrfMRI	Greater functional connectivity of the thalamus to the ACC and left insula associated with stronger VNS efficacy in the responders with a predication accuracy of 86–88%.
Mithani et al., 2019	56	Epilepsy	VNS	NA	NA	NA	25 ± 23	DTI MRI MEG	Increased fractional anisotropy in the left thalamocortical, limbic, and association fibers; increased functional connectivity in the left thalamic, insular, and temporal areas in the responders with a prediction accuracy of 83–90%.

Some of the data are provided as mean ± standard deviations. This table includes two different studies on the same trial (Smith et al., 2012; Sankar et al., 2015)

**Fig. 1:**
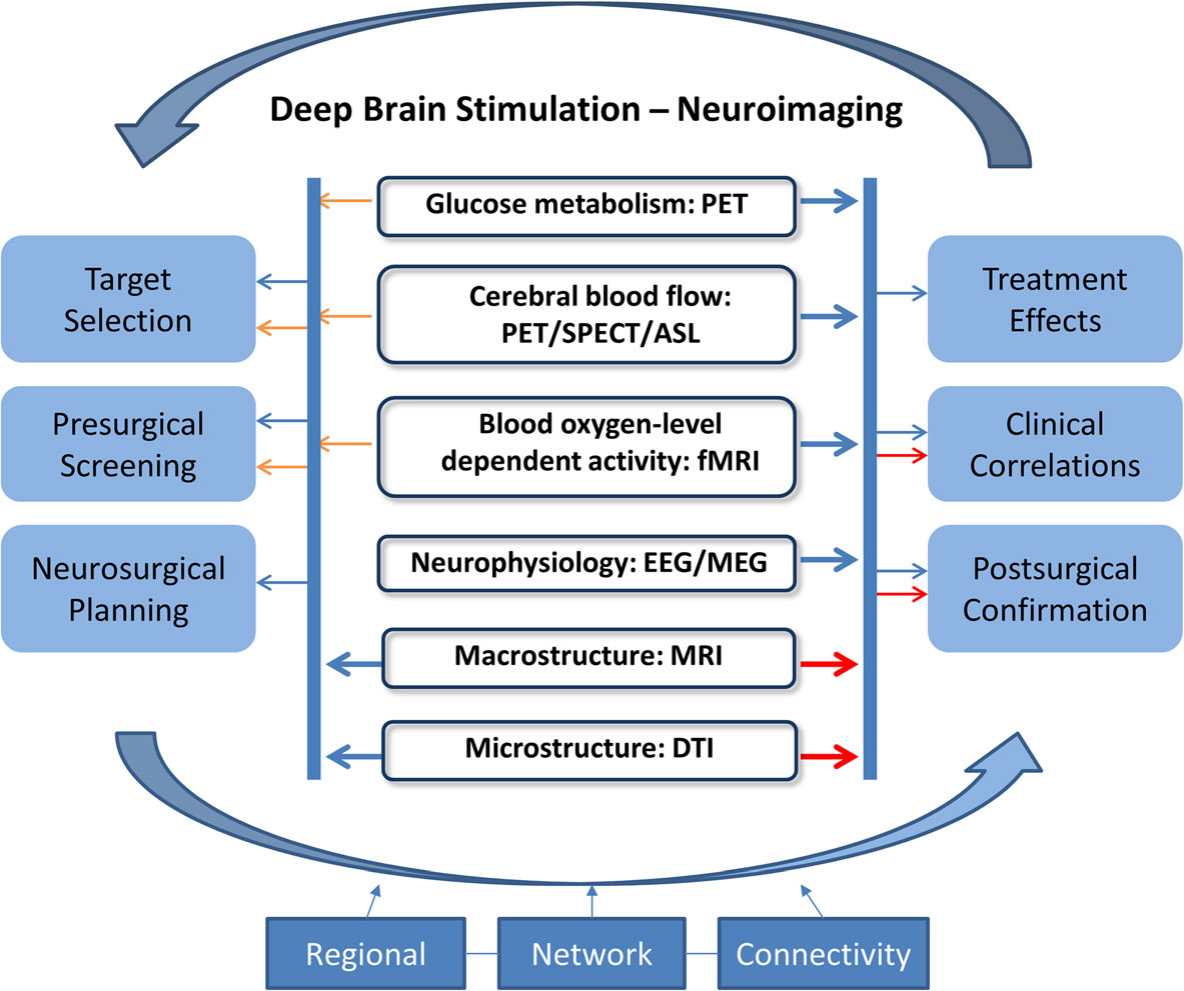
A schematic to illustrate the synergistic interactions between neurosurgical interventions of brain stimulation and multimodal neuroimaging methods of functional and anatomical markers. It also showcases the big feed-back loop and unique organic relationships among different domains that together drive the innovation in the implementation of this therapy in the context of clinical trial design and translational research. The major roles played specifically by individual techniques are represented using the same colors with greater contributions denoted by thicker lines

Multimodality neuroimaging techniques at resting-state have played significant roles in the successful implementation of DBS therapy as illustrated in a flow diagram (Fig. 1). Preoperative MRI/CT has been instrumental in guiding the process of neurosurgical planning and assisting in the operation of DBS implantation. It is fortunate that MRI can be performed safely during DBS neurosurgery or stimulation with commercially available electrodes that are MRI-compatible (Horn et al., 2019, Gratwicke et al., 2020). Indeed, multi-series structural images from intraoperative MRI/CT are valuable to ascertain the accuracy of lead placement of electrodes and assess potential postoperative complications such as acute hemorrhage and edema (Fenoy et al., 2017). Preoperative and postoperative metabolic F18-fluorodeoxyglucose (FDG) PET, perfusion SPECT and structural/functional MRI are commonly employed to provide multimodality neuroimaging correlates of clinical response and improvement (Peng et al., 2014a, Suetens et al., 2014, Dougherty et al., 2016, Lozano et al., 2016, Fridgeirsson et al., 2020, Furukawa et al., 2020). Electroencephalography (EEG) and magnetoencephalography (MEG) can also detect DBS-induced dynamic modulation in large-scale cortical brain networks with high temporal resolution (Kibleur and David, 2018, Mithani et al., 2019, Widge et al., 2019, Litvak et al., 2020). Treatment-related changes in imaging variables and their correlations with clinical outcome measures can be evaluated objectively from regional and network perspectives with the applications of advanced brain mapping methods. Emerging novel analytical approaches based on human connectome can further help reveal mechanisms of action from altered functional and anatomical connectivity between local and remote brain regions of interest (Coenen et al., 2020, Horn and Fox, 2020, Treu et al., 2020).

## Deep brain stimulation (DBS)

DBS is the most prevalent example of symptomatic interventions in movement disorders and other neurological and psychiatric conditions (McKinnon et al., 2019, Sullivan et al., 2021). Recent developments of long-lasting and rechargeable battery to power implantable pulse generator (IPG) and precise segmental electrodes can improve patient comfort (Holland et al., 2020) and enable on-demand delivery of stimulation based on closed-loop designs (Bouthour et al., 2019). Stimulation of neuroanatomic targets in central and peripheral nerve systems is believed to modulate neuronal activity locally and remotely in order to restore circuitry involved in normal brain functioning. This form of reversible and adjustable neuromodulation therapy is made possible with innovations in brain stimulation technology and applications of structural and functional neuroimaging. In particular, there exists a synergistic relationship (see Fig. 1): lessons learned from neuroimaging research become driving forces to further refine every component of stimulation therapy which in turn provides impetus for more neuroimaging studies to understand the mechanisms of improved clinical efficacy.

Presurgical MRI is helpful in ruling out patients with other silent structural lesions and vascular complications in individual brains. It can also help optimize the trajectories of neurosurgery in light of individual variation in target anatomic structures that are small in three dimensions. Of note, MRI with DTI has generated the tractography of cortico-basal ganglia and corticothalamic connections for predicting improved outcome of DBS for neurological and psychiatric disorders (Baldermann et al., 2019, Mithani et al., 2019, Coenen et al., 2020, Horn and Fox, 2020, Treu et al., 2020). A variety of statistical brain mapping software based on univariate and multivariate statistical analytical methods are freely available online. These techniques have been widely used to assess DBS-mediated functional and anatomic changes and their relationships with degrees of clinical improvement in individual patients.

## Movement disorders

Movement disorders represent a variety of neurodegenerative diseases with progressive motor and cognitive dysfunction involving cortico-striato-pallido-thalamocortical (CSPTC) circuits. DBS at many different deep nuclei structures has been most successful in moderate to advanced patients with Parkinson’s disease (PD) for whom available medications are no longer effective (Limousin and Foltynie, 2019). This can bring sustained clinical improvement in motor symptoms with much less drug-related complications and dyskinesia as a result of reduced dose in medications. DBS effects are system-specific regardless of any particular structure stimulated on the circuit. Every structure in the CSPTC system can be targeted (c.f., Fig. 4), i.e. internal globus pallidus (GPi), subthalamus (STN) and putamen to improve bradykinesia/rigidity and thalamus to suppress tremor (Milosevic et al., 2018, Al-Fatly et al., 2019, Vitek et al., 2020). Implantation of DBS electrodes is facilitated by MRI-guided neurosurgery in conjunction with intraoperative microelectrode recording and microstimulation trials.

**Fig. 2:**
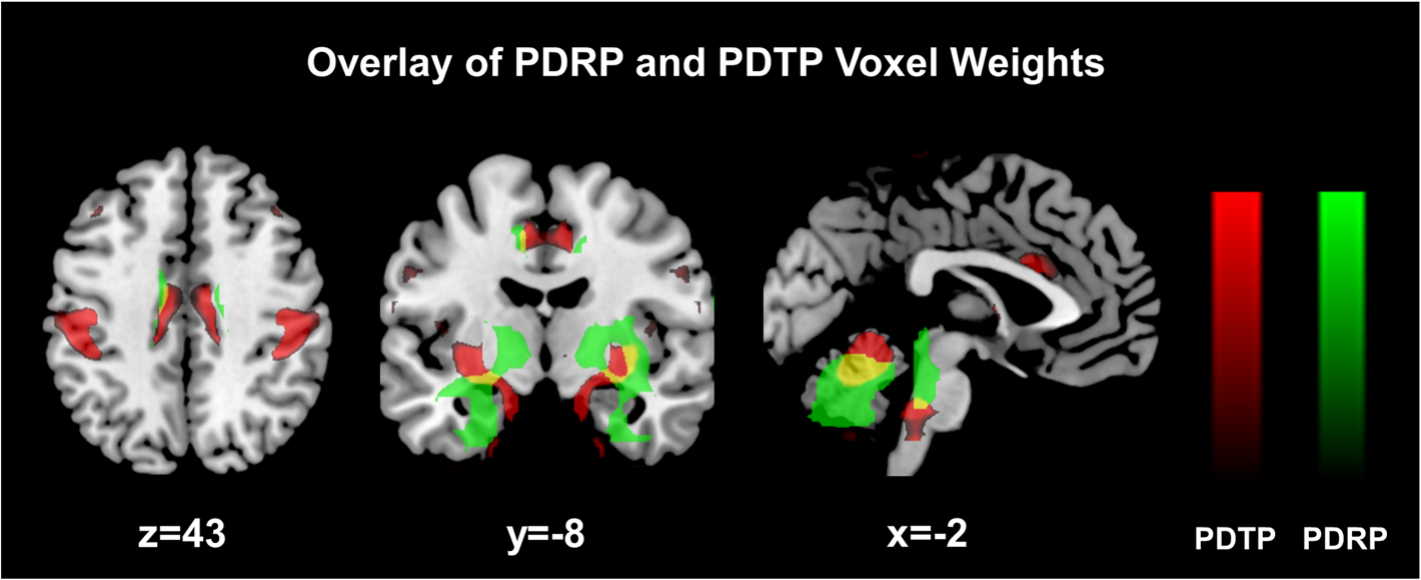
Comparison of PDRP and PDTP topographies. Display of brain areas contributing to PDRP (*green*) and PDTP (*red*) metabolic networks along with areas of overlap (*yellow*) primarily in cerebellum, pons and putamen. The images for both patterns were overlaid on a standard MRI brain template and share 18% of variance between all non-zero voxel weights. [Reprinted from Neuroimage 54:1244–1253. Mure H, Hirano S, Tang CC, Isaias IU, Antonini A, Ma Y, Dhawan V, Eidelberg D. Parkinson’s disease tremor-related metabolic network: characterization, progression, and treatment effects. Copyright (2011), with permission from Elsevier]

CBF and glucose metabolism measured with PET/SPECT has been employed since early 2000s to study experimental therapies with DBS in PD (Peng et al., 2014a), well before the invention of electrodes that can be safely operated in an MRI environment. Changes in brain function and correlation with clinical outcome measures provided by the unified Parkinson’s disease rating scale (UPDRS) motor scores can be determined using both regional and network analytical approaches. To this end, FDG PET has played a crucial role particularly with the application of two unique metabolic brain patterns associated with motor and tremor in PD (Mure et al., 2011, Peng et al., 2014b, Schindlbeck et al., 2020). These are called PDRP and PDTP respectively (see Fig. 2). PDRP is described by increased metabolic activity in the putamen/pallidum, thalamus, sensorimotor cortex, pons, and cerebellum, associated with decreased activity in the premotor and posterior parieto-occipital regions. PDTP is characterized by increased metabolic activity in the primary motor cortex, cerebellum/dorsal pons, and the caudate/putamen. Both patterns reflect widely-distributed network abnormality in the CSPTC and cerebello-thalamo-cortical pathways respectively. It has been reported that DBS at STN and GPi suppresses the expression of PDRP whose changes can predict clinical outcomes, in keeping with the findings of lesioning procedures at the same targets and medical therapy with L-DOPA (Peng et al., 2014a). These results disclose common mechanisms of metabolic brain network modulation and confirm that metabolic effects are less pronounced in DBS than in ablative lesioning.

**Fig. 3:**
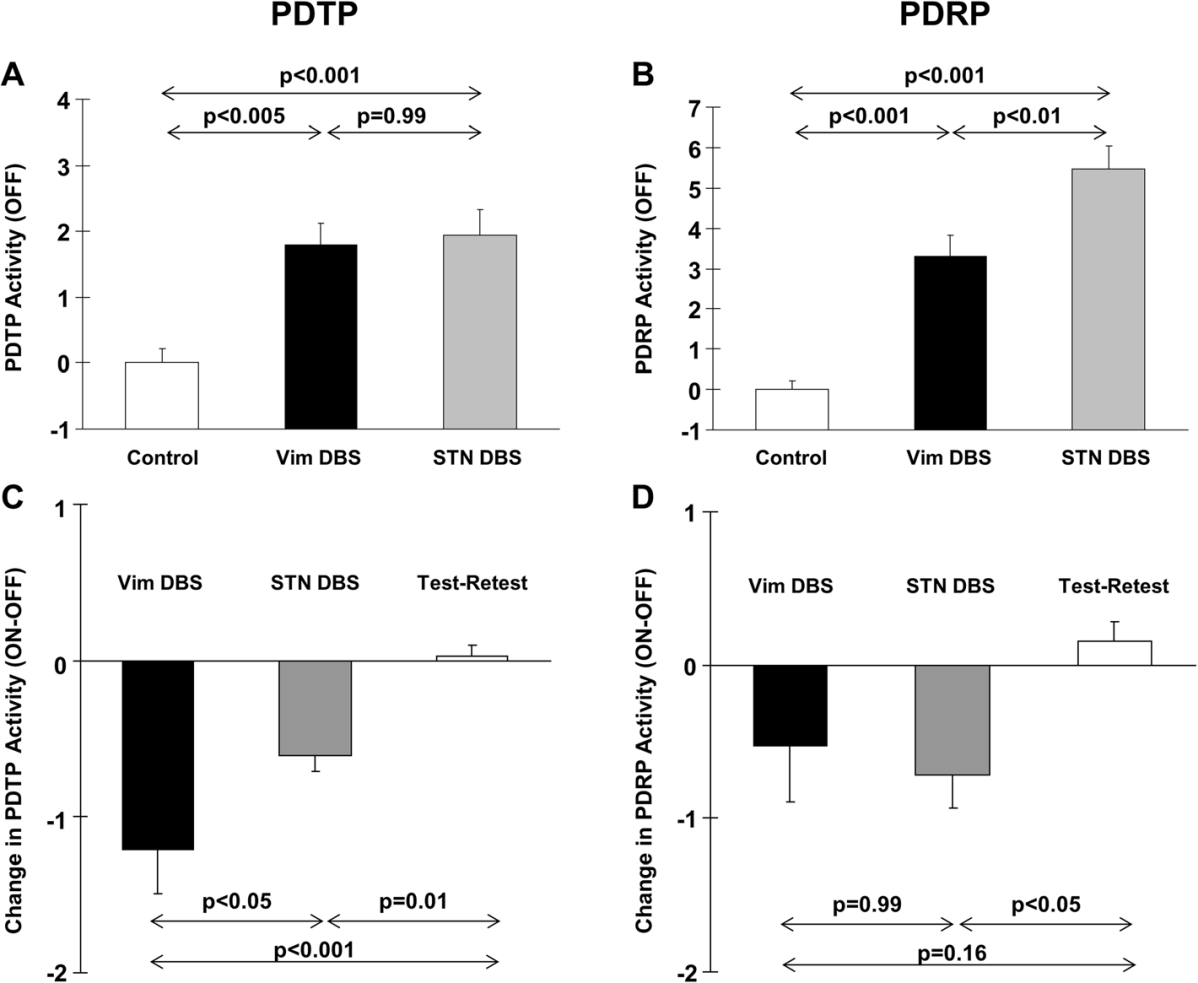
Mean baseline and changes in metabolic network activity of PDTP/PDRP with deep brain stimulation in PD. **a/b**. Baseline activity of both networks was elevated in the Vim STN and the STN DBS groups relative to normal controls. PDTP activity was comparable between the two DBS treatment groups although PDRP activity was higher in the STN than in the Vim DBS group (*p* < 0.01). **c/d**. STN DBS reduced activity of both networks compared to the test-retest PD group (*p* < 0.05) while Vim DBS changed PDTP activity (*p* < 0.001) but did not alter PDRP activity. PDTP activity was suppressed more by Vim DBS than by STN DBS (*p* < 0.05). [Data are presented as mean and standard error; Reprinted from Neuroimage 54:1244–1253. Mure H, Hirano S, Tang CC, Isaias IU, Antonini A, Ma Y, Dhawan V, Eidelberg D. Parkinson’s disease tremor-related metabolic network: characterization, progression, and treatment effects. Copyright (2011), with permission from Elsevier]

We have investigated differences in brain network modulation by ventral intermediate (Vim) thalamic and STN DBS in patients with PD using PDTP and PDRP (Mure et al., 2011). Subject scores of network activity were computed on a hemispheric basis in nine tremor-dominant patients with Vim DBS (seven unilateral and two bilateral) and nine other typical patients with bilateral STN DBS (Table 1). Baseline PDTP/PDRP activity differed among Vim and STN DBS patients and 20 healthy control subjects (*p* < 0.001; one-way ANOVA; Fig. 3: A/B). Elevation in baseline activity relative to controls was comparable in both treatment groups for PDTP (*p* < 0.005) but higher in the STN than in the Vim DBS group for PDRP (*p* < 0.01). Treatment-mediated changes (ON–OFF) in network activity in the same Vim and STN DBS patients and 14 test-retest PD control subjects (Fig. 3: C/D) were also different across the three groups (PDTP: *p* < 0.001; PDRP: *p* = 0.02; one-way ANOVA). Compared with the test-retest PD group, PDTP network activity declined in both DBS groups (Vim: *p* < 0.001; STN: *p* = 0.01) with greater PDTP modulation with Vim than with STN DBS (*p* < 0.05). Conversely, PDRP expression were reduced with STN DBS (*p* < 0.05) but not with Vim DBS. These studies demonstrated that differential effects of novel antiparkinsonian interventions on different motor features of PD can be evaluated objectively by changes in activity of specific metabolic brain networks.

**Fig. 4:**
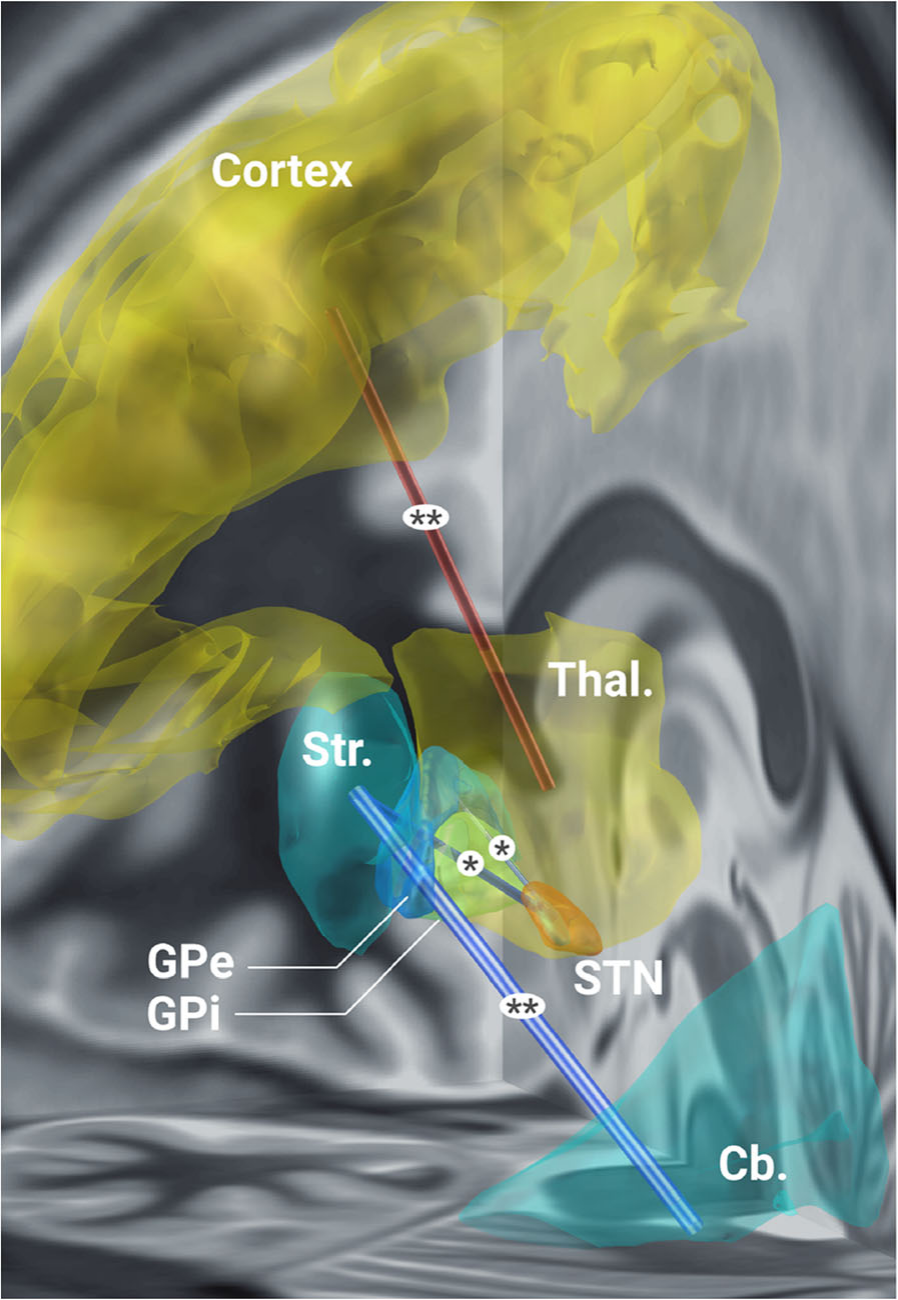
Modulation of specific connections in the motor network by effective STN DBS. Functional connectivity increased between motor thalamus and motor cortex as a function of DBS impact on motor STN. Connectivity decreased between the motor STN and motor striatum or motor external globus pallidus (GPe) and between the motor striatum and cerebellum [**p < 0.005, *p < 0.05 corrected for multiple comparisons using the network-based statistical analysis; Horn A, Wenzel G, Irmen F, Huebl J, Li N, Neumann WJ, Krause P, Bohner G, Scheel M, Kuhn AA. Deep brain stimulation induced normalization of the human functional connectome in Parkinson’s disease. Brain 2019; 142:3129–3143, by permission of Oxford University Press]

In recent years, rsfMRI has been employed to measure changes in voxel-wise connectivity between the target to other structures on intrinsic brain networks found to be abnormal in PD. In a study of 20 patients treated with STN DBS (Horn et al., 2019), local impact of DBS on the motor STN accounted for half the variance in global connectivity increases within the motor network and could estimate the degree of the connectivity normalized towards healthy controls (Table 1). DBS also attenuated a PD-specific network by increasing coupling between motor thalamus and motor cortex but decreasing striatal coupling with cerebellum, external pallidum and STN (Fig. 4). This approach has proven to be very helpful in understanding how DBS modulates the functional connectome of the brain.

Baseline MRI analysis and volumetric measures can help optimize patient selection and stimulation setting and provide valuable prognosis on clinical outcomes. For example, positions of DBS electrodes and volumes of stimulation at different targets determined from post-operative MRI can predict clinical efficacy in PD (Horn et al., 2017, Treu et al., 2020) and dystonia (Schonecker et al., 2015, Reich et al., 2019). The clinical outcomes of DBS may be negatively influenced by underlying brain atrophy and other issues which are often present in PD patients with cognitive complaints at more advanced stages. One preoperative MRI study showed that the cortical thickness of paracentral area and superior frontal region predicted clinical improvement in 31 patients with non-demented PD following STN DBS (Muthuraman et al., 2017). A higher stimulation voltage was needed for an optimal clinical response in patients with cortical atrophy. In addition, cingulate and thalamic volumes were related with robust motor response to STN DBS in 86 patients with PD (Younce et al., 2019, Yim et al., 2020). Of interest, hippocampal volumes were smaller in 14 patients who subsequently developed dementia compared to 14 matched patients who were stable after STN DBS (Aybek et al., 2009). Patients with a rapid onset of dementia following DBS showed significant higher white matter lesion volumes compared to cognitive normal and non-demented patients (Blume et al., 2017). This volume was associated to the rate of cognitive decline within 3 years after DBS surgery. Hence, regional brain atrophy and white matter lesion are potential risk factors for poor motor response or onset of dementia In patients undergoing STN DBS.

A recent study reported impaired cognitive function in 21 patients with PD following bilateral STN DBS with SPECT perfusion imaging (Furukawa et al., 2020). Despite improved motor symptoms and intact general cognition in the whole group there was a decline in the drawing ability in half of the patients (52%) after the treatment. Comparing DBS with the preoperative baseline, CBF in the PD group decreased in the bilateral dorsolateral prefrontal cortex (DLPFC) (BA 8/9) and anterior and middle cingulate cortex (BA 24/32), but increased in the left precuneus, parieto-temporal cortex (BA 7, 19, 39, and 40) and cerebellum (Table 1). Specifically, CBF was lower bilaterally in the middle cingulate cortex or supplementary motor area in patients with declined drawing performance. By contrast, CBF decreased in right DLPFC and increased in left parieto-occipital regions in patients with reserved drawing performance. These findings demonstrate that the decline in drawing ability developed in some PD patients after STN DBS reflects possible dysfunction in the cingulate network.

In summary, multimodality neuroimaging studies with MRI and PET/SPECT have played key roles in the successful delivery of DBS therapy in movement disorders. In particular, functional imaging markers based on PET/SPECT and rsfMRI demonstrate that the central effects of treatment on clinical outcome and cognitive deterioration are found to be associated with of target-specific brain network modulation. Moreover, cortical-subcortical atrophy and white matter lesions revealed by preoperative structural MRI may represent distinct predictors of poor motor outcome and onset of dementia in PD patients after STN-DBS. This type of work provides critical information that can be used by physicians to maximize clinical efficacy but minimize adverse outcomes in each individual patient.

## Dementia

Elderly patients often develop cognitive problems over the course of disease progression in a wide spectrum of neurodegenerative disorders. There is usually a transition from mild cognitive impairment (MCI) to different forms of dementia as late-stage manifestations. In the following section we cover some applications of DBS in the treatment of dementia resulting from PD and AD.

### Dementia in PD

PD dementia (PDD) and dementia of Lewy bodies (DLB) have been described in the literature of parkinsonism depending on the clinical diagnostic criteria. Bilateral DBS of the nucleus basalis of Meynert (NBM) at low frequency has been proposed as a promising therapy for patients with dementia given that this nucleus, populated with abundant cholinergic neurons, is a key relay structure associated with dysfunctional brain circuits of memory and cognition (Koulousakis et al., 2019, Lee et al., 2019). Participants are evaluated before and after NBM DBS according to many clinical outcome measures such as mini-mental state examination (MMSE), Mattis dementia rating scale (MDRS) and comprehensive neuropsychological and neuropsychiatric assessments. Three studies have been reported on this topic with multimodality neuroimaging (see Table 1). Six patients with mild to severe PDD took part in a randomized, double-blind, crossover clinical trial of this intervention over 6 weeks along with rsfMRI to evaluate the effects on functional connectivity (Gratwicke et al., 2018). No clinical improvement was found in NBM DBS versus sham stimulation according to primary cognitive outcomes despite moderate benefits in total scores of Neuropsychiatric Inventory. Both active and sham DBS induced changes in resting-state BOLD activity within the default mode network but did not show any significant differences at the group-level.

Six other patients with mild to moderate DLB underwent the same regimen also did not improve clinically with only a trend of improvement in the severity of neuropsychiatric symptoms (Gratwicke et al., 2020). Active stimulation revealed some changes of functional connectivity in both the DMN and the frontoparietal network. A French study in six patients with mild to moderate DLB reported no significant differences in primary outcomes and other scales between active and sham NBM DBS after 3 months of the treatment (Maltete et al., 2021). Comparing the active NBM DBS to baseline, phonemic fluency and motor disability significantly decreased but superior lingual gyrus metabolic activity increased with FDG PET.

In conclusion, these studies report important pilot trials to demonstrate the safety and tolerability of this intervention and the potential for brain network modulation despite no robust improvement in cognitive function. In addition to continued efforts to individually improve target placement and stimulation setting it would be necessary to replicate the findings in larger samples with longer courses of treatment.

### Alzheimer’s disease

Alzheimer’s disease (AD) is the most common form of neurodegenerative dementia affecting principally elder populations across the world. Of note, MCI represents a high-risk category of prodromal AD given that it falls into intermediate clinical stages between healthy aging and the onset of dementia. The severity of the disease is evaluated quantitatively by many clinical dementia rating scales such as the Alzheimer’s Disease Assessment Scale – Cognitive subscale 13 (ADAS-Cog 13). There is strong evidence for central involvement of widespread brain networks behind memory loss and cognitive dysfunction in AD, but only limited options are available for viable medical treatments. NBM and fornix in the medial forebrain have been explored as potential targets of choice for neurosurgical interventions due to their prominent roles in cholinergic systems (Mirzadeh et al., 2016, Koulousakis et al., 2019, Lee et al., 2019). Four relevant studies have been published on this therapy with the use of neuroimaging methods (Table 1). Indeed, a pilot study of low frequency NBM DBS in six mild to moderate AD patients showed responses in four cases based on primary clinical endpoints over 12 months of treatment along with metabolic changes seen in FDG PET (Kuhn et al., 2015). The trend-level increases in metabolism were detected in the whole brain including the temporal, parietal and amygdalo-hippocampal regions.

An earlier FDG PET study evaluated metabolic responses in five of six AD patients treated with high frequency DBS at the fornix for 12 months in a phase-1 trial (Smith et al., 2012). Connectivity analysis on FDG PET scans revealed that metabolism increased in two orthogonal networks: a fronto-temporo-parieto-striato-thalamic network and a fronto-temporo-parieto-occipito-hippocampal network. The effects of DBS on the cortical circuitry were greater compared with pharmacotherapy. In addition, higher baseline metabolism and treatment-related changes in these regions were predictive of clinical outcomes in global cognition, memory, and quality of life. Of importance, DBS at the fornix in all six AD patients in the preceding study slowed the progression of hippocampal atrophy and hypometabolism over 12 months compared with patients without this treatment using both structural MRI and FDG PET imaging (Sankar et al., 2015). In addition to correlating with changes in hippocampal metabolism, volume changes in the hippocampus correlated strongly with those in the fornix and mammillary bodies (Table 1), reflecting a circuit-wide effects of stimulation on major inflow and output pathways from the hippocampus and medial temporal lobe. DBS also led to volume expansion in other regions known to be atrophic in AD, including the bilateral parahippocampal gyrus, right superior temporal gyrus, left inferior parietal lobule, and bilateral precuneus. This is the first in-human study to show that DBS not only modulates neural circuit metabolic activity but also attenuates the natural course of brain atrophy in a neurodegenerative disease.

A phase-2 trial of this fornix-DBS therapy in 42 patients with mild AD revealed no differences in primary cognitive outcomes in the whole cohort over 12 months (Lozano et al., 2016). In patients with stimulation ON and OFF, glucose metabolism in the pre- and post-central gyri, temporo-parietal association cortices, hippocampus, cuneus, and cerebellum increased at 6 months but not at 12 months (Table 1). Further analysis disclosed a significant interaction between age and treatment outcome. About 28% of the patients younger than 65 years of age become worse while 72% of patients older than 65 years of age showed trend-levels in both clinical improvement and greater increases in regional metabolism. This age effect could be attributed to re-operative PET scans revealing lower metabolism in temporal and parietal areas in the young compared to the old patients despite similar cognitive deficits at baseline. The safety profiles and clinical responses reported in this cohort were confirmed in a follow-up study over 24 months (Leoutsakos et al., 2018).

In summary, these studies confirm that DBS of NBM/fornix in patients with dementia is generally safe and well-tolerated but has brought no overall improvement in cognitive outcomes in these early-stage trials. The clinical results are variable suggesting the need to tailor stimulation parameters on an individual basis for the desired responses. Nevertheless, DBS can consistently reverse the decreased metabolism over the course of AD and resulting cortical metabolic changes have some potential to predict better clinical outcomes in patients. More work is necessary to optimize key aspects of technology before mounting efficacy trials of this therapy.

## Obsessive-compulsive disorders

Obsessive-compulsive disorder (OCD) is one of prevalent psychiatric diseases with abnormal functional and structural substrates involving the CSPTC brain circuitry. Approximately 10% of OCD patients are refractory to first-line treatment and need neurosurgical interventions such as ablative lesioning or DBS. Several structures including ventral capsule/ventral striatum (VC/VS), ventral anterior limb of the internal capsule (ALIC), nucleus accumbens (NAc) and anteromedial STN are effective targets for severe treatment-refractory OCD based on open-label trials (Baldermann et al., 2019, Huys et al., 2019). It is important to understand how these interventions lead to clinical improvement and potential differences in network modulation. PET imaging with FDG and H_2_O has been instrumental in assessing the restoration of abnormal brain function and specific relationships with clinical outcome measures obtained with the Yale-Brown Obsessive Compulsive Scale (Y-BOCS) and the Hamilton Depression Rating Scale (HDRS).

Since its approval by the Food and Drug Administration (FDA) in early 2008 for the treatment of refractory OCD, global efforts have begun to focus on DBS (Graat et al., 2020, Sullivan et al., 2021). In this section we discuss five related neuroimaging studies of interest on this promising therapy (Table 1). In a randomized, double-blind and crossover study, 10 patients with OCD were treated with high-frequency bilateral STN DBS and scanned with FDG PET (Le Jeune et al., 2010). A significant decrease in metabolism was seen in the left cingulate and medial frontal gyri comparing ON and OFF conditions. The improvement in Y-BOCS scores correlated positively with metabolic changes in medial prefrontal and orbitofrontal regions. These early results demonstrate that the therapeutic effect of STN DBS is associated with reduced prefrontal glucose metabolism.

Of great interest, one study compared differences in metabolic network modulation between DBS and capsulotomy at the same target of VC/VS for refractory OCD (Suetens et al., 2014). Two groups of age-matched OCD patients underwent FDG PET at OFF and ON DBS conditions or before and after capsulotomy. Both groups had comparable preoperative clinical characteristic and imaging features. Baseline metabolism in anterior cingulate cortex (ACC) and superior temporal regions predicted postoperative improvement in depressive symptoms. Both interventions resulted in common postoperative metabolic decreases in ACC and prefrontal and orbitofrontal cortices. Compared with DBS, capsulotomy induced greater metabolic changes, with additional significant decreases in mediodorsal thalamus, caudate nucleus, and cerebellum as well as increases in precuneus and fusiform and lingual gyrus. Improvement in Y-BOCS scores correlated with metabolic changes in the occipital cortex. These findings show that DBS and capsulotomy have produced similar clinical improvement and metabolic network changes in core structures both within and outside the CSPTC in neurosurgical treatments in OCD .

A PET study with H_2_O examined the effects of different electrode contacts on CBF in six patients receiving acute high frequency VC/VS DBS (Dougherty et al., 2016). CBF increased in the dorsal ACC with monopolar DBS turned on at the most ventral contact (Table 1). This increase correlated highly with the improvement in depressive symptoms but not with OCD symptoms. CBF also increased in the thalamus, striatum, and globus pallidus with DBS turned on at the most dorsal contact. These findings can be helpful in maximizing clinical efficacy in individual patients.

**Fig. 5:**
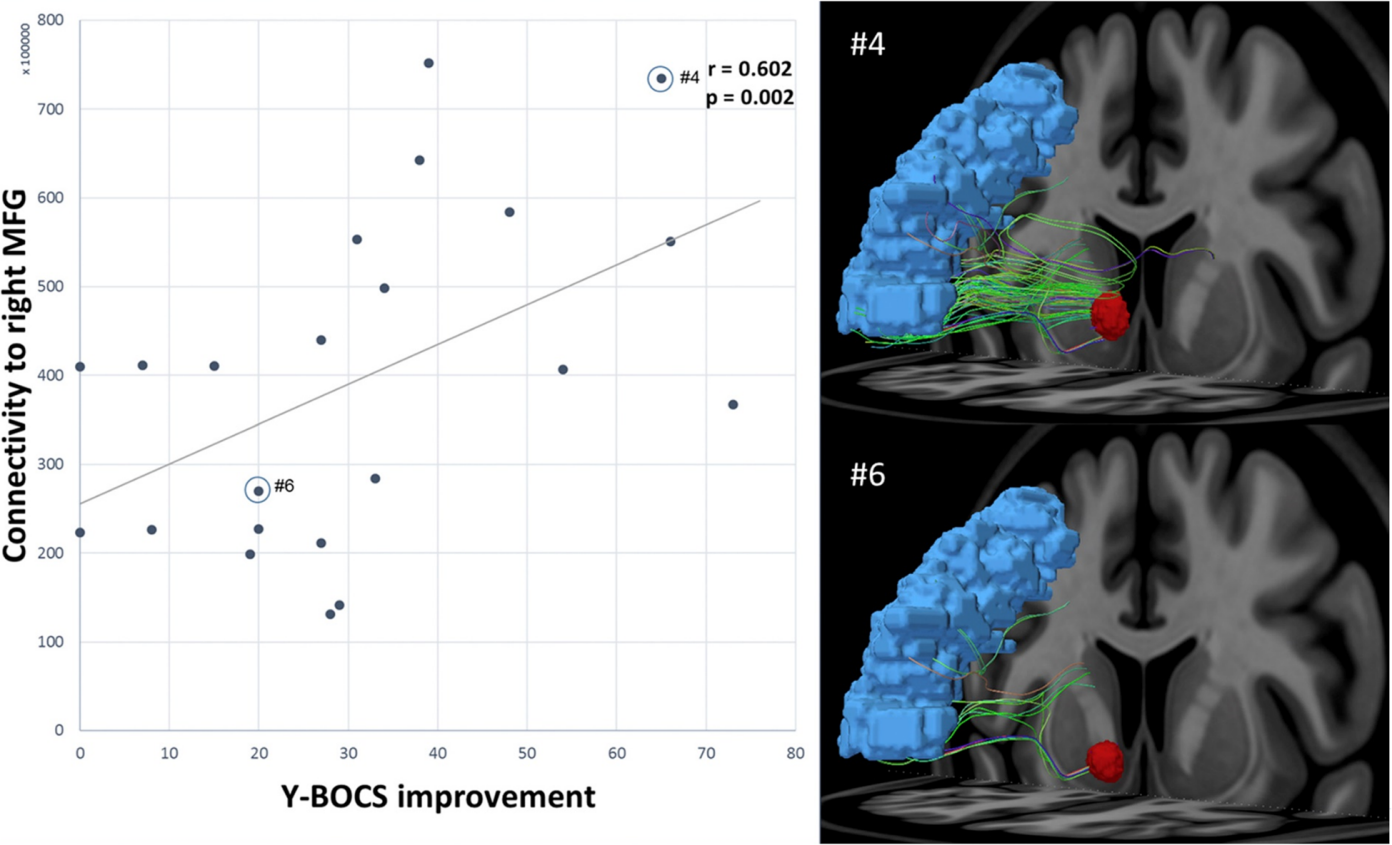
Correlation of clinical outcome and connectivity to cortical regions of interest. Left panel: Connectivity of stimulation sites to the right middle frontal gyrus (MFG) associated positively with clinical outcome after 12 months of DBS. Right panel: Patient 4 exhibited more robust clinical outcome to DBS with greater connectivity to the caudal part of the MFG region. Patient 6 showed less robust clinical outcome to DBS with relatively sparse connectivity. [Reprinted from Biological Psychiatry 85 (9): 735–743, Baldermann JC, Melzer C, Zapf A, Kohl S, Timmermann L, Tittgemeyer M, Huys D, Visser-Vandewalle V, Kuhn AA, Horn A, Kuhn J. Connectivity profile predictive of effective deep brain stimulation in obsessive-compulsive disorder. Copyright (2019), with permission from Society of Biological Psychiatry]

Neuroimaging studies with multimodal MRI methods have also revealed new mechanistic insights on therapeutic responses by examining the effects of DBS on the intrinsic brain connectivity impaired in OCD. One recent study using preoperative DTI identified brain networks involved with clinical outcome measures in 22 patients with OCD who were treated with DBS at the ventral ALIC/NAC (Baldermann et al., 2019). A positive correlation was detected between clinical improvement in Y-BOCS scores and anatomical connectivity of stimulation sites to brain regions involving the medial and bilateral lateral prefrontal cortex (PFC) by tracking fibers connecting with estimated volumes of tissue activated using the normative connectome data available (Table 1). *Post-hoc* analysis confirmed that the positive correlation involved specifically the connectivity to the right middle frontal gyrus (MFG; Fig. 5). Indeed, effective DBS outcome was found to be associated with the fiber track within the ventral ALIC that enters the ventral thalamus connecting with the middle PFC. Further analysis suggested that fiber tracks enclosing the cingulum, ventromedial prefrontal cortex (vmPFC) and fornix were predictive of improvement in depressive symptoms. This study shows that specific anatomical connectivity maps from DTI tractography data can be quantified to provide reliable predictors of clinical outcomes prior to participating in DBS therapy in a single patient with OCD.

Another study with rsfMRI reported the modulation of directional connectivity in the limbic system in 10 patients with OCD treated by ventral ALIC DBS for at least 12 months (Fridgeirsson et al., 2020). DBS decreased the functional connectivity between amygdala and insula to the level comparable to that in healthy subjects (Table 1). DBS-related changes in connectivity correlated positively with changes in anxiety and mood. Further analysis of directionality showed that the impact of vmPFC over the amygdala increased in patients during DBS ON compared to DBS OFF and to healthy controls. By contrast, the impact of amygdala on insula decreased in the patients during DBS ON compared to DBS OFF and the heathy controls. Th increased and decreased values indicate excitatory and inhibitory connections respectively. These results highlight the importance of the amygdala circuit in the pathophysiology of OCD and explain how DBS at this target can bring sustained clinical improvement in patients.

In conclusion, these studies confirm that DBS at different targets have produced similar clinical improvement and robust metabolic network changes in the CSPTC circuit with a prominent role for the ACC. This view is supported by a recent report with intraoperative MRI/DTI demonstrating that VC was connected primarily to the medial orbitofrontal cortex (OFC), with anteromedial STN projected mainly to the lateral OFC, dorsal ACC, and DLPFC (Tyagi et al., 2019). The studies with multimodal neuroimaging can help identify clinical responders in advance and shed lights on important roles that cortical regions outside this circuitry may play in symptomatology of OCD and related psychiatric disorders.

## Vagus nerve stimulation

In recent years, peripheral interventions based on vagus nerve stimulation (VNS) have emerged as a viable form of neuromodulation therapies (Carreno and Frazer, 2017, Johnson and Wilson, 2018). This technology can bring clinical improvement in patients with various psychiatric disorders by modulating neuronal activity in remote target structures in the brain given that vagus nerves have widespread connections with the brain and other organ systems in the body (Pavlov, 2019). Indeed, electronic stimulation of these nerve endings and related projections has been a promising strategy with numerous neuroimaging studies published using both invasive and noninvasive therapeutic approaches. Here we focus on the use of multimodality neuroimaging methods to investigate the efficacy of invasive VNS for the treatment of medication-resistant depression and epilepsy.

### Major depression

Neuroimaging has been valuable in assessing the therapeutic responses in medically intractable patients with major depression (MD) undergoing VNS (Table 1). An open-label European multi-center study reported on safety and efficacy of low frequency VNS in 15 patients with MD using perfusion SPECT (Kosel et al., 2011). After 10 weeks of VNS, CBF increased in the left dorsolateral/ventrolateral prefrontal cortex (BA 46 and 47), but decreased in the right posterior cingulate area, the lingual gyrus and the left insula. This was in line with earlier reports that chronic VNS increased CBF in the left DLPFC, a key area involved in the neurobiology of depression. The modulation of the activity in this region could be responsible for the antidepressant efficacy of VNS. One FDG PET study examined associations between acute and chronic antidepressant VNS responses and changes in regional metabolism in 13 patients with MD (Conway et al., 2013). Metabolism in nine VNS responders (i.e., 12-month HDRS reduced by ≥50%) decreased in the right DLPFC (BA 9), dorsomedial prefrontal cortex (BA 9), rostral ACC (BA 32) at 3 months from baseline. CBF also decreased in the right superior temporal gyrus (BA 42) and right posterior insular cortex at 3 and 12 months, but increased in the left inferior temporal gyrus and cerebellum at 12 months. Moreover, metabolism increased in the left ventral tegmental area at 3 and 12 months with changes correlated significantly with improvement in HDRS scores. This effect of antidepressant response was mediated through the locus coeruleus (Grimonprez et al., 2015). These results suggest that VNS response may involve brain plasticity from subacute to chronic VNS even in the presence of psychotropic medications.

### Epilepsy

VNS is also one of major brain stimulation modalities to treat medically refractory epilepsy but with highly variable rates of responses in both adults and children (Foit et al., 2020). Patients are commonly designated as clinical responders and non-responders defined as ≥50 and < 50% reduction in seizure frequency after the procedure. Despite its valuable roles in evaluating VNS-mediated brain network modulation, neuroimaging techniques are critically important in helping identify the responders who are most likely to benefit from this invasive intervention particular in pediatric populations (Table 1). One study analyzed preoperative FDG PET data in 61 pediatric patients with epilepsy who were followed up for a minimum of 12 months after VNS (Yu et al., 2018). There was a significant difference in metabolic connectivity between 35 responders and 26 non-responders. Relative changes in glucose metabolism were less connected in brainstem, cerebellum, putamen, cingulate gyrus and bilateral insula in the responders compared to the non-responders.

Other imaging modalities have also been employed to select suitable candidates before initiating VNS therapy. One study acquired preoperative rsfMRI data in 21 children with epilepsy who were mostly treated for at least 12 months with VNS (Ibrahim et al., 2017). See-based analysis disclosed that increased functional connectivity from the thalamus to the ACC, vmPFC and left insula was associated with greater VNS efficacy. The clinical responders were identified with an accuracy of 86% in the training sample of 21 patients and 88% in a small testing sample of another eight patients using a support vector machine (SVM) learning classifier with rigorous cross-validation. These results agree with a recent multi-center study which evaluated 56 epileptic children with preoperative DTI and rest-state MEG (Mithani et al., 2019). This cohort was treated by VSN for more than 24 months on average and divided in a training sample of 38 patients and a testing sample of 18 patients. Treatment responders of 27 patients showed greater fractional anisotropy in left thalamocortical, limbic, and association fibers than the non-responders. DTI-guided analysis of MET data in a subset of subjects revealed greater connectivity in a functional network involving the left thalamic, insular, and temporal nodes. The responders could be predicated with an accuracy of 90% in the training sample and 83% in the testing sample using the same classification scheme. The accuracy of predication was significantly superior using dual-modality imaging markers compared with multiple clinical variables.

In summary, robust changes in specific pathways and associated connectivity revealed by multimodal neuroimaging methods further support the notion that VNS is able to bring symptomatic relief by modulating distinct brain networks underlying these disorders. In particular these multi-institutional studies demonstrate the power of connectome in providing new insights into the mechanism of action for VNS. The novel classification algorithms based on neuroimaging data are highly accurate in predicting treatment responses in individuals and prevent some patients from participating in invasive and costly VNS procedures.

## Development of new frontiers

### Innovations in technology

Brain stimulation has been an effective option in treating a variety of neurodegenerative and neuropsychiatric disorders with deep and peripheral brain stimulation (Graat et al., 2020, Horn and Fox, 2020, Sullivan et al., 2021). There have been two major hurdles associated with brain stimulation technology. Firstly, IPG had limited battery lives and had to be replaced surgically and reprogrammed after about 5 years. This drawback was mitigated by the development of rechargeable implantable pulse generators to increase patient comfort, reduce long-term cost for DBS and eliminate the surgery for IPG placement (De Vloo et al., 2018, Hitti et al., 2018, Holland et al., 2020). Secondly, the efficacy of this intervention had been variable depending on accurate placement of electrodes on specific targets and precise setting of stimulation paradigms for each individual patient with a particular disorder. This was evident in many early-stage clinical trials with neuroimaging where smaller numbers of patients had been treated by DBS using different stimulation sites/parameters (Table 1). Bioelectronic computational models of DBS based on DTI MRI brain scans had also been evaluated to rapidly select patient-specific settings in mobile devices (Janson and Butson, 2018). Recent technological advances in the design of high precision segregated electrodes and wearable monitors allowed for real-time adjustment of these variables automatically based on closed feedback loops (Bouthour et al., 2019). Clinical need of each patient could be balanced by engaging multi-target stimulation (Tyagi et al., 2019) and multi-frequency programming (Karl et al., 2020). The continued advances in these areas will ultimately result in optimal care of patients with personalized medicine.

### Challenges in clinical trial design

There have been multiple obstacles in translating promising therapeutic solutions from preclinical to clinical arenas in neurological disorders. Firstly, different functional biomarkers were used for the mechanistic assessment of efficacy in light of inevitable inter-species differences. This situation could be overcome by developing in animal models viable imaging biomarkers that have proven effective in patient populations for use in preclinical trial of novel therapeutics. A primary example had been the work with FDG PET on metabolic brain networks in non-human primate models of experimental parkinsonism undergoing striatal dopamine cell implantation (Ma et al., 2015, Peng et al., 2016). That approach represented a form of inverse translation in which preclinical studies of neurosurgical interventions replicated brain network modulation analogous to that reported in clinical trials. Secondly, many phase-2 clinical trials had failed to reach primary end points because of robust placebo responses in patients receiving sham treatments. Such patients could be screened individually with preoperative FDG PET scans in trial candidates by assessing the expression of a sham surgery-related metabolic brain network (Ko et al., 2014). Thirdly, clinical trial design had been suboptimal by including patients with inaccurate clinical diagnosis at the study entry. This deficiency needs to be addressed rigorously by using a variety of specific neuroimaging markers and novel statistical methods.

The last decade has witnessed the development and clinical validation of large-scale metabolic brain networks involving widely-distributed functional neuroanatomical structures. This was especially true with FDG PET in patients with PD and atypical PD such as multiple system atrophy, progressive supranuclear palsy and cortico-basal degeneration (Teune et al., 2013, Niethammer et al., 2014, Peng et al., 2014b, Ge et al., 2018, Schindlbeck and Eidelberg, 2018, Meles et al., 2020, Shen et al., 2020). Novel classification algorithms based on the expression levels of these networks provided accurate differential diagnosis even in early-stage parkinsonian populations (Tang et al., 2010, Tripathi et al., 2016), given that DBS was ineffective in pathologically-confirmed patients with atypical PD who were inadvertently misdiagnosed to receive the invasive treatment (Meissner et al., 2016). Of special note, this network-based diagnostic method had helped exclude about 11% patients referred by movement disorders specialists to participate in a phase-2 gene therapy trial in PD delivered at subthalamus (LeWitt et al., 2011), the same target as used for DBS in PD. It is this critical step of prescreening that was likely responsible for the successful outcomes among many failed gene therapy trials.

Studies with FDG PET had also reported robust metabolic brain networks associated specifically with cognitive dysfunction in PD (Meles et al., 2015, Mattis et al., 2016), AD (Mattis et al., 2016, Meles et al., 2017) and fronto-temporal dementia (Nazem et al., 2018). These brain network patterns were expressed differently in patients with MCI and various forms of dementia and also highly reproducible across multiple medical centers. Expression scores of these brain networks provided proven ability for differential diagnosis and correlate with clinical measures of disease severity. This promising approach has not yet been widely used in clinical trials of brain stimulation therapy for symptomatic relief in neurodegenerative patients suffering from cognitive complaints.

Recent studies in clinical trials suggest a need for early-interventions to delay onset or slow down progression of the disease. Work is currently under way to develop DBS as potential neuroprotective therapies to intervene early in the disease course (McKinnon et al., 2019). Neuroimaging is expected to help overcome additional challenges to identify patients before the onset of clinical symptoms. For example, expression of PDRP was reported to be elevated in patients with rapid eye movement sleep behavioral disorder who subsequently converted to PD (Holtbernd et al., 2014). Expression of PD cognition-related brain pattern (PDCP) was also elevated increasingly in PD patients with MCI and PDD (Mattis et al., 2016). It is currently not known whether prognostic or treatment outcome could also be assessed by PDRP/PDCP generated by perfusion imaging with ASL MRI (Melzer et al., 2011, Teune et al., 2014) and resting-state activity with rsfMRI data (Wu et al., 2015, Vo et al., 2017). A recent study established a unique metabolic brain network related with phenoconversion from MCI to AD using a large sample of FDG PET images available in public domain (Blazhenets et al., 2019). Analogous brain network of cerebral atrophy could also be created in concurrently acquired structural MRI data. Altogether, prognostic analysis based on the use of these brain networks can help select prodromal subjects destined to develop MCI and dementia in PD and AD.

### Advances in multimodal neuroimaging

Multimodality neuroimaging techniques have accelerated the pace of development and evaluation of stimulation-based therapeutics by using various analytical approaches showcased in this review. To this end we have cited many examples demonstrating the unique advantages of multimodal neuroimaging data. It is known that anatomical imaging is indispensable for target identification and neurosurgery while functional imaging is mostly useful for probing brain responses in relation to clinical outcome measures (Table 1). In general, brain metabolism obtained with FDG PET has superior signal-to-noise ratio (SNR) than CBF measured with perfusion PET/SPECT and ASL MRI. Comparable to a large degree among themselves, these imaging markers have greater SNR but lower spatial-temporal resolution than rsfMRI used to map brain functional connectivity. In addition, there is always inevitable interaction or overlap between measurements of functional markers and brain atrophy inherent in neurodegenerative disorders. Although integrating multimodal neuroimaging data has been proven to be more powerful the choice of any particular combination of imaging modality is always dictated by questions of interest and practical considerations such as the availability and trade-off between sample size and cost. The last decade has seen the remarkable technological transformation of hybrid imaging systems from powerful PET/CT cameras to more advanced PET/MRI scanners. This innovation opens the unprecedented avenues to fully probe functional and anatomical substrates in patients and animal models simultaneously under identical neurophysiolological conditions. In addition to valuable advantages of performing concurrent multi-modality neuroimaging more economically, it also affords a viable platform to examine the engagement of molecular targets and the restoration of brain circuitry simultaneously.

One interesting development in the field of molecular imaging is the availability of PET radioligands that are specific to microglial activation. This has been used successfully to detect neuroinflammation in cortical and subcortical regions in intervention studies and neurological disorders such as PD, AD and epilepsy (Gershen et al., 2015, Jucaite et al., 2015, Sandiego et al., 2015, Kreisl et al., 2017). Modulation of neuroinflammatory response by DBS has been suggested as a potential therapeutic strategy in animal models of neurodegenerative diseases (Campos et al., 2020, Chen et al., 2020) although this has not been demonstrated in patient populations. Nevertheless, VNS has been shown to impact immunity in the brain and many other organ systems upstream and downstream the site of stimulation (Pavlov, 2019). Time has come to explore in patients or animal models with PET imaging how DBS or VNS may influence neuroinflammation in relation to changes in brain network markers and clinical outcomes.

One of the most revolutionary areas in neuroimaging research that drive the development of DBS-based therapeutics is the recent applications of human connectome and associated software tools based on DTI and rsfMRI data (Coenen et al., 2020, Horn and Fox, 2020, Treu et al., 2020). Numerous studies have shown that target-specific anatomic and functional connectivity profiles can reliably predict clinical outcomes of DBS in a variety of neurological disorders such as PD (Horn et al., 2017), essential tremor (Al-Fatly et al., 2019), OCD (Baldermann et al., 2019), depression (Coenen et al., 2020) and epilepsy (Mithani et al., 2019). The ability to identify the responders in advance of neurosurgical intervention can greatly improve patient care especially in vulnerable populations like children who might otherwise be exposed to anesthetic and surgical risks without any clinical benefits. This approach has also been used to evaluate network modulation and clinical correlation by DBS in several disorders (Horn et al., 2019, Fridgeirsson et al., 2020, Gratwicke et al., 2020). These studies confirm the power of connectome to bring unique insights on the therapeutic mechanisms of DBS by mapping changes in local connectivity in large-scale brain networks characteristic of each disease entity.

## Conclusion

A number of internal and external brain stimulation modalities have been approved by FDA for providing symptomatic relief in many neurological and psychiatric disorders. This mode of intervention is most effective when combined with prevalent pharmacotherapy without major complications but generally unable to halt progression of the underlying disease. There is still only a limited number of randomized, double-blind and placebo-controlled trials of DBS over long-term despite proven clinical efficacy in many trials over short-term. Multimodal neuroimaging methodology has successfully guided the development this fledgling field from the beginning, demonstrated its translational potential and brought critical insights on mechanisms of action. This is achieved by revealing specific functional neuroanatomical substrates associated with each disorder, optimizing neurosurgical procedures, tailoring stimulation paradigms, identifying clinical responders and detecting modulation of brain function on regional and network levels in human trials. There is still a urgent need to develop and cross-validate robust neuroimaging markers and diagnostic algorithms for use in multi-center clinical trials both nationally and internationally. Applications of such maturing and innovative techniques will continue to play pivotal and integral roles in further validating and advancing this revolutionary field of bioelectronic medicine.

## Data Availability

Data sharing is not applicable to this article as no datasets were generated or analyzed during the current study.

## Abbreviations

ACC: Anterior cingulate cortex
AD: Alzheimer’s disease
ADAS: Alzheimer’s disease assessment scale
ALIC: Anterior limb of internal capsule
ASL: Arterial spin labeling
BOLD: Blood-oxygen-level-dependent
CBF: Cerebral blood flow
CSPTC: Cortico-striato-pallido-thalamo-cortical
CT: Computerized tomography
DAT: Dopamine transporter
DBS: Deep brain stimulation
DLB: Dementia with lewy bodies
DLPFC: Dorsolateral prefrontal cortex
DTI: Diffusion tensor imaging
ECD: Ethyl cysteinate dimer
EEG: Electroencephalography
FDA: Food and drug administration
FDG: Fluorodeoxyglucose
GPe: External globus pallidus
GPi: Internal globus pallidus
HDRS: Hamilton depression rating scale
HMPAO: Hexamethyl propylene amine oxime
IMP: Iodoamphetamine
IPG: Implantable pulse generator
LD: Levodopa
MCI: Mild cognitive impairment
MD: Major depression
MDRS: Mattis dementia rating scale
MEG: Magnetoencephalography
MFG: Middle frontal gyrus
MMSE: Mini-mental state examination
MRI: Magnetic resonance imaging
MSA: Multiple system atrophy
NAC: Nucleus accumbens
NBM: Nucleus basalis of meynert
NIBS: Non-invasive brain stimulation
OCD: Obsessive-compulsive disorder
OFC: Medial orbitofrontal cortex
PCA: Principal component analysis
PD: Parkinson’s disease
PDCP: PD-related cognitive pattern
PDD: Parkinson’s disease dementia
PDRP: PD-related covariance pattern
PDTP: PD tremor-related pattern
PET: Positron emission tomography
PFC: Prefrontal cortex
PMC: Premotor cortex
PSP: Progressive supranuclear palsy
rsfMRI: Rest-state functional MRI
SMA: Supplementary motor area
SMC: Sensorimotor cortex
SNR: Signal-to-noise ratio
SPECT: Single photon emission computed tomography
STN: Subthalamic nucleus
SVM: Support vector machine
UPDRS: Unified parkinson’s disease rating scale
VC/VS: Ventral capsule/ventral striatum
Vim: Ventral intermediate
vmPFC: Ventromedial prefrontal cortex
VNS: Vagus nerve stimulation
Y-BOCS: Yale-brown obsessive compulsive scale
